# Supplementary material for: Somatic Mutations in Latin American Breast Cancer Patients: A Systematic Review and Meta-Analysis
Source: Diagnostics (Basel). 2024 Jan 29;14(3):287. doi: 10.3390/diagnostics14030287 (PMC10855727; doi:10.3390/diagnostics14030287)
Supplement: Supplementary file 1 [file diagnostics-14-00287-s001.zip › Supplementary Figure S1.pdf]

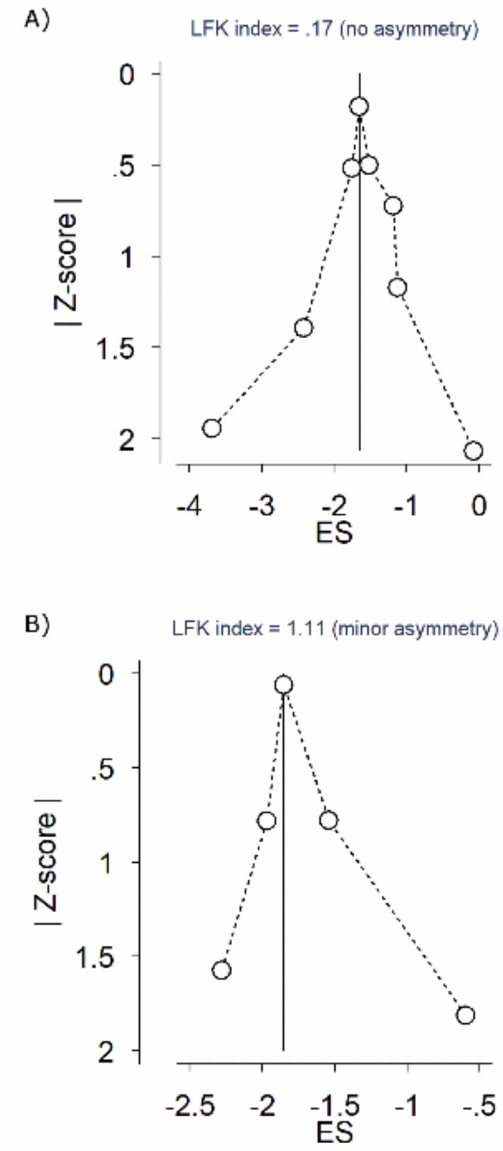


**Supplementary Figure S1**. Doi plot and LFK index for proportions of mutations in (A) *TP53* and (B) *PIK3CA* reported in Latin American patients´ tumors.
