# Supplementary material for: Somatic Mutations in Latin American Breast Cancer Patients: A Systematic Review and Meta-Analysis
Source: Diagnostics (Basel). 2024 Jan 29;14(3):287. doi: 10.3390/diagnostics14030287 (PMC10855727; doi:10.3390/diagnostics14030287)
Supplement: Supplementary file 1 [file diagnostics-14-00287-s001.zip › Supplementary Figure S2.pdf]

A)

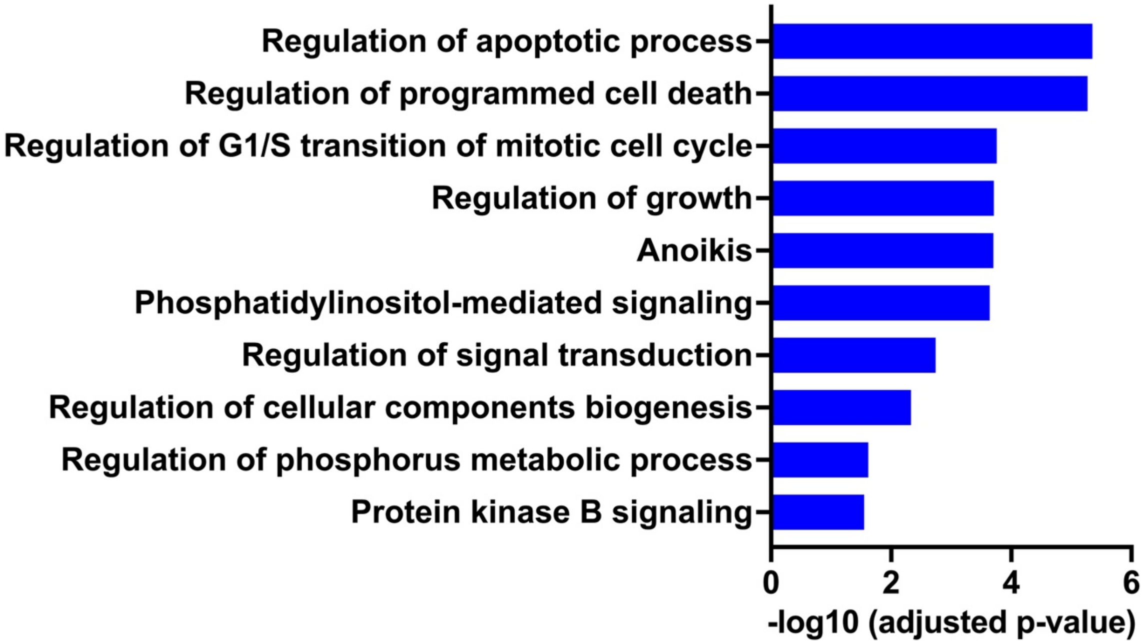

B)

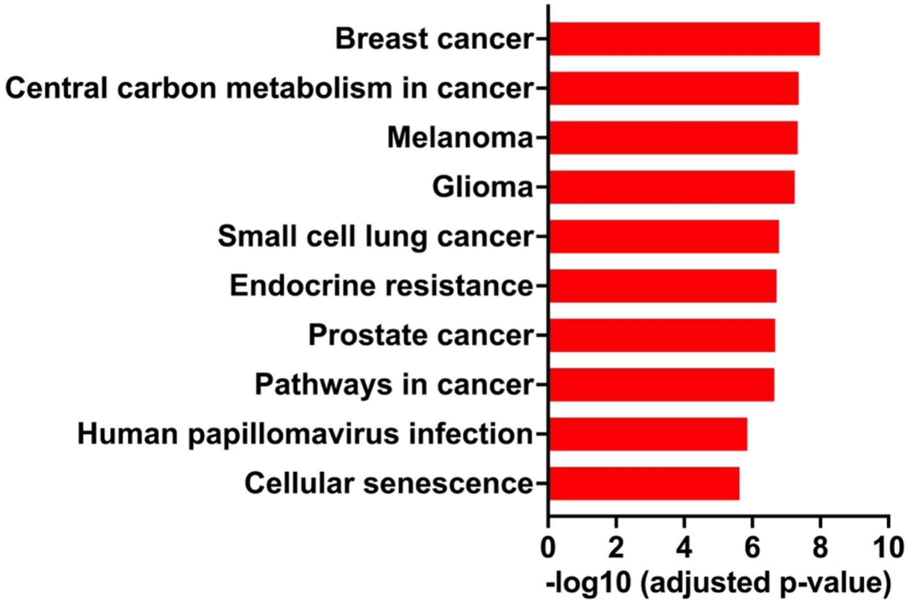

Supplementary Figure S2. (A) Main non-redundant biological processes overrepresented and (B) top ten of enriched canonical pathways, using the list of seven genes mutated in the molecular subtype TNBC.
