# Supplementary material for: Somatic Mutations in Latin American Breast Cancer Patients: A Systematic Review and Meta-Analysis
Source: Diagnostics (Basel). 2024 Jan 29;14(3):287. doi: 10.3390/diagnostics14030287 (PMC10855727; doi:10.3390/diagnostics14030287)
Supplement: Supplementary file 1 [file diagnostics-14-00287-s001.zip › Supplementary Figure S4 120124.pdf]

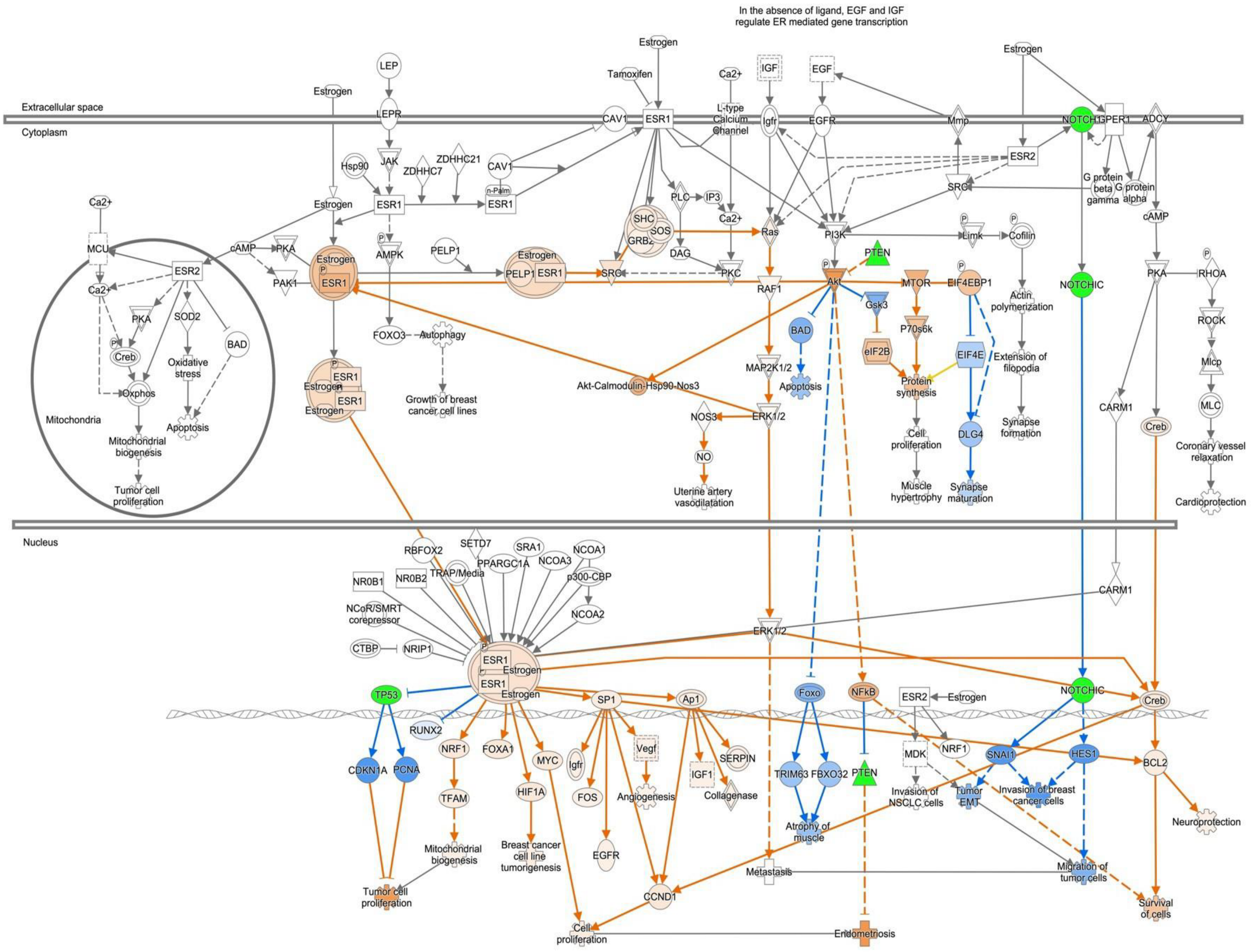

© 2000-2023 QIAGEN. All rights reserved.

Supplementary Figure S4. Molecular activity prediction analysis of the canonical pathway estrogen signaling defined by the Ingenuity Knowledge Base. Three common downregulated genes (*TP53*, *PTEN*, and *NOTCH1*) with loss of function mutations in both molecular subtypes of breast cancer [TNBC and HER2(+)] modify several bio-functions associated with the progression of this disease.
