# Supplementary material for: Somatic Mutations in Latin American Breast Cancer Patients: A Systematic Review and Meta-Analysis
Source: Diagnostics (Basel). 2024 Jan 29;14(3):287. doi: 10.3390/diagnostics14030287 (PMC10855727; doi:10.3390/diagnostics14030287)
Supplement: Supplementary file 1 [file diagnostics-14-00287-s001.zip › Supplementary Table S1 .pdf]

**Table S1.** Type of somatic mutation found in reported genes in patients from Latin America.

| Mutation Type | Gene N (%) | | | | | | | | |
|---|---|---|---|---|---|---|---|---|---|
| | *TP53* | *PIK3CA* | *FLT3* | *AKT1* | *CDKN2A* | *CDH1* | *PTEN* | *RB1* | *NOTCH1* |
| | (n=200) | (n=87) | (n=16) | (n=12) | (n=7) | (n=6) | (n=6) | (n=5) | (n=3) |
| **Synonymous** | 12 (6.0) | 7 (8.05) | | | 1 (14.29) | | | | |
| **Missense** | 107 (53.5) | 61 (70.1) | | 12 (100) | 5 (71.43) | 1 (16.67) | | 2 (40.0) | 3 (100) |
| **Nonsense** | 20 (10.0) | 1 (1.15) | | | | | 2 (33.33) | 2 (40.0) | |
| **Deletion** | 7 (3.5) | | | | | | 2 (33.33) | | |
| **Frameshift** | 23 (11.5) | | | | | 5 (83.33) | 2 (33.33) | 1 (20.0) | |
| **In frame** | 2 (1.0) | | | | | | | | |
| **Insertion** | 2 (1.0) | 1 (1.15) | | | 1 (14.29) | | | | |
| **Intronic** | 26 (13.0) | 16 (18.4) | 16 (100) | | | | | | |
| **Stop codon lost** | | 1 (1.15) | | | | | | | |
